# Asylums for the Deaf and Dumb

**Published:** 1840

**Authors:** 


### EDUCATION OF THE DEAF AND DUMB.

In natural connexion with the foregoing case, we are led to notice our western institutions for the education of deaf mutes. They are two in number, the older in Danville, Kentucky, the younger in Columbus, Ohio. The former, however, seems to have attracted less attention, and to have grown less rapidly than the latter, and we have not at hand, any recent accounts on its condition.

"*The thirteenth annual Report of the Trustees of the Ohio Asylum for the year* 1839," has, however, been sent to us and must receive a moment's notice.

The number of its pupils is seventy-two—of which, at this time, one is from Pennsylvania, one from Michigan, one from Indiana and one from Louisiana, all the rest from Ohio. The population of that state is estimated at a million and a half, which gives about 22,700 to each of the scholars, sent by her to this school; but the proportion of deaf mutes must be much greater, as there is reason to believe that not a fifth part of that unfortunate class are sent thither. In the catalogue for 1839, seventy-two names are printed, of which we find that forty-one are males and thirty-one females. This remarkable difference, must not be received as evidence that there are in the State more deaf male, than female children—except so far as there are more male than female births; for a greater proportion of the former than of the latter are sent from home to be educated. Appended to the name of each pupil, is the name of the disease, as far as it can be made out, which occasioned the deafness, and the reader will perhaps share in our surprise, at learning that but twenty-six, less than one-third of the whole, were born deaf—of the rest, ten are marked as uncertain or unknown, which added to the congenital deaf make exactly one-half of the aggregate. Of the twenty-six congenital mutes, fourteen are males and twelve females; of the ten marked as unknown, but classed by us with the natural deaf mutes, six are males and four females. But let us take up those in whom the deafness was the manifest offspring of disease after birth. These make thirty-six or one-half of the whole, and may be classed as follows: Inflammation, dropsy and other affections of the head, eleven—fever, eight—sickness, six—colds, four—and small-pox, measles, scarlatina, whooping-cough, cutaneous disease, swellings under the jaw, and coffee in the external ear, each one—thirty-six, of which twenty-one are males and fifteen females. Thus it appears, that while the number of the congenital mutes is, in reference to the two sexes, nearly the same, or as fourteen to twelve, the number of deaf males from disease, is to the other sex, as twenty-one to fifteen. If an equal proportion of the two sexes were sent to the Asylum, these statistics would show, that more boys than girls become deaf, from the diseases of childhood, which indeed, we should, a *priori*, expect to be the case, inasmuch as they are more exposed to the remote causes of those acute diseases of the head which so often terminate in this infirmity. At this point of our little analysis, we cannot forego one or two practical observations.

*First.* The excessive use of animal food by indulgent parents, who make no distinction between the constitutions of children and adults, is unquestionably one cause of the frequency of inflammatory affections of the head.

*Second.* The use of caps, and warm hats and bonnets, in infancy and childhood, is another custom contributing though in a less degree to the same result.

*Third.* The omission of blood-letting, in the cerebral, and catarrhal diseases of the same class of patients, when the symptoms require it, because of the difficulty of performing the operation, is another and most prolific cause of the same sinister effects. If the aggregate of the children, lost or permanently injured by this omission, could be made out, the catalogue would be appalling. How many die of inflammation of the brain and its membranes, who might be saved by the timely use of the lancet! How many tedious ulcerations of the external auditory passages might not be warded off by the same treatment! What is the ear-ache, the great scourge of childhood nine times out of ten, but an inflammation, which is treated, ten times out of eleven, with stimulants and narcotics!

The following paragraph from the report of Mr. Hubbell, the teacher will be read with interest by physiologists:

"The most important item of information respecting our Ohio Deaf and Dumb, that has been elicited since the publication of our last annual report, is, the existence of a large number of mutes in a particular neighborhood in Highland county. Their number is sixteen, and are found between the ages of two and fifty, though they are almost between the ages of three and twenty. They were all born deaf, and are six males and ten females. They are all so intricately connected by marriage and birth that it is difficult to describe their consanguinity. They are all uneducated, of German descent, and no applications have ever been made for the admission of any of them into the Deaf and Dumb Asylum. For the above information I am indebted to the politeness of David Fenwick, Esq., of Mourytown, Highland county."

It is no part of c r plan to give an account of the methods of instruction and discipline pursued in this Asylum, which is so honorable to the State which founded and sustains it, but having seen much of its internal economy and the progress of its pupils, we must be allowed to express our very high approbation of both. The superintendent and principal instructor, Mr. Hubbell, is not only a gentleman of great urbanity, but devoted heart and soul to his duties. D.

